# Unacylated ghrelin modulates circulating angiogenic cell number in insulin-resistant states

**DOI:** 10.1186/s13098-017-0239-8

**Published:** 2017-05-31

**Authors:** Behiye Özcan, Pieter J. M. Leenen, Patric J. D. Delhanty, Lucy Y. Baldéon-Rojas, Sebastian J. Neggers, Aart Jan van der Lely

**Affiliations:** 1 Department of Internal Medicine, Erasmus MC, Rotterdam, The Netherlands; 2 Department of Immunology, Erasmus MC, Rotterdam, The Netherlands

**Keywords:** Diabetes, Circulating angiogenic cell, Unacylated ghrelin, Insulin resistant, Obese

## Abstract

**Background:**

Type 2 diabetes (T2D) is associated with reduced numbers and impaired function of circulating angiogenic cells (CAC) which contributes to the progression of atherosclerosis and microvascular disease. Previous studies suggest that short-term infusion of unacylated ghrelin (UAG) normalizes CAC number in patients with T2D. To determine dose-dependent effects of short-term infusion of UAG in T2D patients using a cross-over model, and of long-term infusion of UAG in obese mice, on differentiation of monocyte progenitors into CAC.

**Methods:**

Eight overweight T2D patients were infused overnight with 3 and 10 µg/kg/h of UAG in a double-blind, placebo-controlled cross-over study. To assess the effects of long-term UAG treatment, obese mice were infused with UAG for 4 weeks. Monocyte progenitors were assessed for their ability to differentiate into CAC in vitro.

**Results:**

In T2D patients, UAG treatment caused a reduction in differentiation of CAC, dependent on UAG dose and differentiation method. However, mice treated with UAG showed a significant increase in differentiation of bone marrow progenitors into CAC.

**Conclusion:**

UAG causes a minor suppressive effect on CAC development after short-term treatment in humans, but experiments in mice suggest that long-term treatment has beneficial effects on CAC formation.

The Netherlands Trial Register: TC=2487

## Background

Ghrelin is a 28 amino acid peptide that is secreted predominantly by X/A-like cells in the stomach. It circulates in two different forms: acylated ghrelin (AG) and unacylated ghrelin (UAG; also named des-acyl ghrelin) [1, 2]. AG is acylated with octanoic acid at serine 3 by ghrelin *O*-acyl transferase (GOAT), whereas UAG is not [3]. Ghrelin was discovered in 1999 as the endogenous ligand of the orphan growth hormone secretagogue receptor (GHSR1a) that had previously been identified as the receptor mediating the pharmacological effects of a new therapeutic class of compounds, the growth hormone secretagogues [4]. Ghrelin was initially characterized for its property of inducing growth hormone (GH) secretion, a function mediated by GHSR1a [4].

Today, however the ghrelin system is known to exert numerous biological effects on the secretion of several pituitary hormones, on gastric acid secretion and gut-motility, on exocrine and endocrine pancreatic function and glucose metabolism, appetite stimulation and the cardio-vascular and immune system [2, 5]. Since UAG only activates GHSR1a at supra-physiological concentrations [6], and has no physiological effect on GH secretion [7], it has long been considered not to have a physiological role.

Clinical evidence suggests that UAG prevents the diabetogenic effects of AG in healthy volunteers and in GH-deficient patients [2, 8], primarily by improving insulin-sensitivity. Importantly, we have recently shown that UAG treatment rapidly improves insulin sensitivity in type 2 diabetic subjects [9]. How it can exert effects in several disparate (patho)physiological systems and conditions remains to be answered. In metabolically active tissues of GHSR-deficient mice, UAG rapidly modulates lipogenic and insulin signaling pathway gene expression [10], indicating a GHSR-independent mechanism of action, although a receptor for UAG remains to be discovered. Recently, we have also shown that UAG infusion in obese mice causes insulin-sensitizing effects [11].

Vascular complications are the major source of morbidity and mortality among patients with diabetes [12]. Diabetes mellitus is associated with a 2- to 4-fold increase in rates of macrovascular disease [13]. This is due to macrophage accumulation in the vascular wall and subsequent secretion of multiple proinflammatory growth factors and cytokines, a very important step in the pathological mechanism causing atherosclerosis [14].

Monocytes and monocyte-derived cells are important in detecting and repairing vascular damage, in particular by producing proangiogenic factors which promote neovascularization and endothelial repair. These vessel-protective cells have previously been indicated as myeloid EPC [15, 16], but are now more accurately termed pro-angiogenic cells or circulating angiogenic cells (CAC). We will use the latter term throughout this study. CAC are involved in restoration of vascular homeostasis in diabetics. Monocyte differentiation and function in patients with diabetes is altered by metabolic conditions, resulting in the decreased ability of these cells to participate in vascular maintenance and repair [17–19]. Data from recent experiments on CAC suggest that UAG may beneficially impact the vascular remodeling process which is known to be impaired in type 2 diabetes patients [20, 21].

The aims of the current study are to extend the findings of Togliatto et al. by examining the dose and time-dependent effects of in vivo UAG treatment on in vitro differentiation of isolated monocytes into CAC using different protocols, and into their presumed non-protective counterparts, macrophages. To do this, we first examined the effects of a short-term, i.e. overnight infusion of UAG at 3 and 10 µg/kg/h in T2D patients on differentiation of peripheral blood monocytes into CAC and macrophages. Since long-term UAG treatment of humans is not ethically feasible, we infused lean and obese mice with vehicle or UAG at 10 µg/kg/h for 4 weeks and examined their effect on differentiation of CAC from bone marrow monocyte progenitors.

## Methods

### Study drug

UAG was obtained from Bachem AG, Hauptstrasse 144, Bubendorf CH-4416, Switzerland. UAG was delivered as lyophilized powder (vials containing 5 mg of drug) and stored as indicated by the manufacturer. The same peptide preparation was used for both animal and human experiments.

## Human peripheral blood mononuclear cell cultures

### Study design

This study is a single-center, investigator-initiated, double blind and placebo-controlled randomized study [9]. The study was conducted in Erasmus University Medical Center Rotterdam after approval by the local IRB. All subjects gave their written informed consent before enrollment. The study included three overnight hospital admissions. During these three admissions, all subjects received either placebo, or UAG at 3 or 10 μg/kg/h in randomized order as a continuous infusion via an indwelling catheter as described below. A washout period of at least 1 week was used between the different treatment periods.

Placebo or UAG was continuously infused for 15 h from 22:00 h until 13:00 h the next day. At 8:00–8:15 h the following morning, a standard breakfast meal (SBM) was served: 714 kcal (46% fat, 17% proteins, 37% carbohydrates). Before the start of each infusion period a continuous glucose monitor (iPro2, Medtronic trading, The Netherlands) was inserted to measure the subcutaneous interstitial blood glucose [22–25].

Blood samples for isolating monocyte progenitors were collected before (16:00) and after (the next day at 14:00) the infusion period.

### Subjects

Eight subjects were enrolled (2 females–6 males; mean age 53 years (range 31–65 years). Mean body mass index (BMI) was 31.5 kg/m^2^, range 26–40.1 kg/m^2^. All subjects were diagnosed with type 2 diabetes for at least 3 months prior to enrollment. Mean glycosylated hemoglobin levels (HbA1c) were 49 (41–55) mmol/mol [6.6 (5.9–7.2)%]. Exclusion criteria were a history or presence of long-term type 2 diabetes complications; clinically significant abnormalities in laboratory evaluation at screening, and the use of systemic corticosteroids within 60 days prior to screening.

Four of eight patients were medicated with statins and two with antihypertensive drugs. All patients were being treated with metformin that was stopped 1 day prior to the start of each treatment period.

### Peripheral blood mononuclear cell cultures

Peripheral blood mononuclear cells (PBMC) were obtained by density gradient centrifugation (Ficoll-Paque PLUS, GE Healthcare 17-1440-03) of 50 ml of peripheral blood collected in sodium heparin tubes. CAC, also called early endothelial progenitor cells (EPC) or myeloid EPC, were cultured according to two different protocols as described by Murohara et al. and by Asahara et al. [26, 27]. These cultures are referred to as Toyo-CAC and EBM-CAC, respectively.

In both cultures, PBMC were plated in triplicate at a density of 2 × 10^6^ cells/ml/well in 24-well plates (Nunc) coated with 10 μg/ml of human fibronectin (Sigma). For Toyo-CAC, cells were cultured in M199 medium supplemented with 10% FCS, 0.05 mg/ml bovine pituitary extract (Invitrogen), 10,000 U penicillin/ml, 10,000 U streptomycin/ml (Lonza DE17-602E), and 10 units/ml heparin (Leo Pharma BV) at 37 °C and 5% CO_2_. For EBM-CAC, cells were cultured in EBM-2 + EGM-2 (Lonza 3156; 4176) at 37 °C and 5% CO_2_. After 7 days of culture, Toyo-CAC and EBM-CAC cultures were washed profusely with PBS to retain only the adherent spindle-shaped cells. Cells were then fixed with paraformaldehyde (PFA, 4%) for 15 m at room temperature.

To generate human macrophages from monocytes under similar conditions, total PBMC were cultured in triplicate at a density of 1 x 10^6^ cells/well at 37 °C and 5% CO_2_ in 24-well plates for 7 days in RPMI (25 mM Hepes, with Ultraglutamin-1) supplemented with human AB serum (Lonza 14-490E), 10,000 U penicillin/ml, 10,000 U streptomycin/ml (Lonza DE17-602E), and 10% M-CSF-containing conditioned medium from Ladmac cells [28]. Fresh growth factor (100 μl) was added to each well on days 3 and 6. At day 7, plates were centrifuged at 1200 rpm for 3 min at 4 °C and cells were fixed with PFA (4%).

The quantification of cultures was performed fluorometrically on the basis of DNA content as assessed by propidum iodide staining [29]. Briefly, wells were incubated with 20 μl propidium iodide (4 μl/ml; Sigma). After 10 min the fluorescence intensity was measured using a FLUOstar Optima plate reader (BMG Labtech, Ortenberg, Germany). Interpolation on a standard curve of PBMC was used to determine absolute cell numbers.

### Differentiation of CAC from bone marrow of lean and obese mice infused with UAG

C57BL/6 mice (10 week, male; Charles River Laboratories, Maastricht, The Netherlands) were maintained at 12:12 h light: dark, 21 °C, with free access to food and water. Normal (ND) and high fat (HFD) diets were from special diets services (Tecnilab-BMI, Someren, The Netherlands; product code 801722, 9% kcal from fat, 69% from carbohydrate, 22% from protein) and ABDiets (Woerden, The Netherlands; 41% kcal from fat, 40% kcal from carbohydrate, 19% kcal from protein), respectively. Animal experiments were performed with the approval of the Animal Ethics Committee at Erasmus MC (approval code: EMC2050). Mice were implanted with subcutaneous Alzet 1004 mini-osmotic pumps (Durect Corp., Cupertino, CA, USA) containing saline or UAG. UAG was infused at 10 µg/kg/h. Mice were split into two groups 2 weeks after pump implantation (n = 3 for each of the 4 experimental groups), either continuing to receive the ND or given free access to the HFD, as previously described [11]. At the end of the 4 week infusion period, bone marrow was collected from the femurs and tibiae, and culture of CAC/endothelial progenitor cells was performed as previously described [30].

Weight and food intake were recorded 1 week before and weekly during the period of treatment. Data on weight, fat mass, glucose and insulin levels in these mice are described in Delhanty et al. [11].

## Results

### Assessment of dose-dependent effects of UAG in humans

**Fig. 1:**
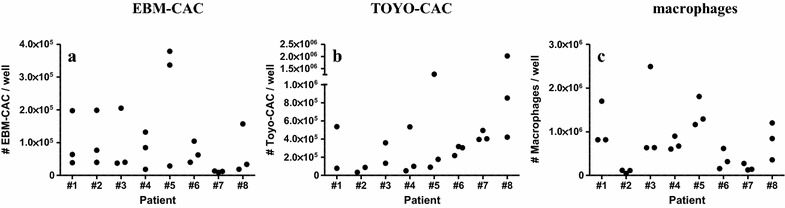
Baseline values for the generation of Toyo-CAC, EBM-CAC, and macrophages. PBMC were isolated from patients at admission, before treatment, and cultured under different conditions to generate EBM-CAC (**a**), Toyo-CAC (**b**), or macrophages (**c**). Each of the three data-points per patient represents the average of three replicate wells

**Fig. 2:**
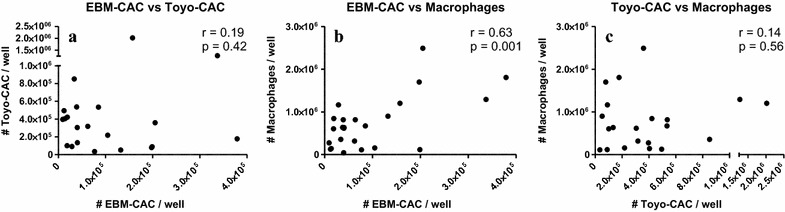
Correlation between EBM-CAC, Toyo-CAC and macrophage baseline culture yields. Outcomes of EBM-CAC, Toyo-CAC and macrophage cultures were correlated. A significant correlation was observed only between EBM-CAC and macrophage cultures (**b**), but not between the two types of CAC cultures (**a**), or the Toyo-CAC and macrophages (**c**)

**Fig. 3:**
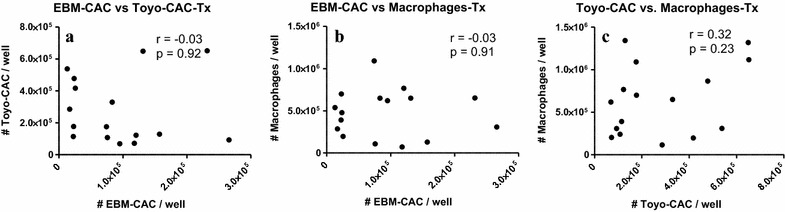
Correlation between EBM-CAC, Toyo-CAC and macrophage culture yields after UAG treatment. Outcomes of EBM-CAC, Toyo-CAC and macrophage cultures from PBMC obtained after treatment with UAG were correlated. No significant correlation was observed between the two types of CAC cultures (**a**), between EBM-CAC and macrophage cultures (**b**), or the Toyo-CAC and macrophages (**c**)

Baseline values of generated EBM-CAC, Toyo-CAC and macrophages are shown in Fig. 1, representing the retrieval of these cell types from cultures of PBMC obtained before the initiation of treatment. In both types of CAC cultures as well as in the macrophage cultures, a significant degree of variation was observed with respect to the absolute number of CAC and macrophages retrieved. This variation occurred not only between different patients, but also among different samples from the same patients obtained at different visits. Baseline culture results from some patients were relatively consistent, while results from others showed high degrees of variation, suggesting a strong influence of variable patient conditions upon admission. Furthermore, cells from some patients showed a virtually complete inability to generate either type of CAC and/or macrophages. For instance, cells from patient #7 generated virtually no EBM-CAC and macrophages, while Toyo-CAC could be readily measured. Another patient #2 generated virtually no Toyo-CAC or macrophages, but EBM-CAC could be shown, though at variable levels. These baseline findings also indicate that both types of CAC cultures and macrophage cultures measure essentially distinct parameters. This is confirmed by a correlation analysis of the culture yields before and after treatment (Figs. 2, 3). This shows that a significant correlation exists only between the quantity of EBM-CAC and macrophages at baseline (Fig. 2b), but this correlation is lost after treatment with UAG (Fig. 3b).

**Fig. 4:**
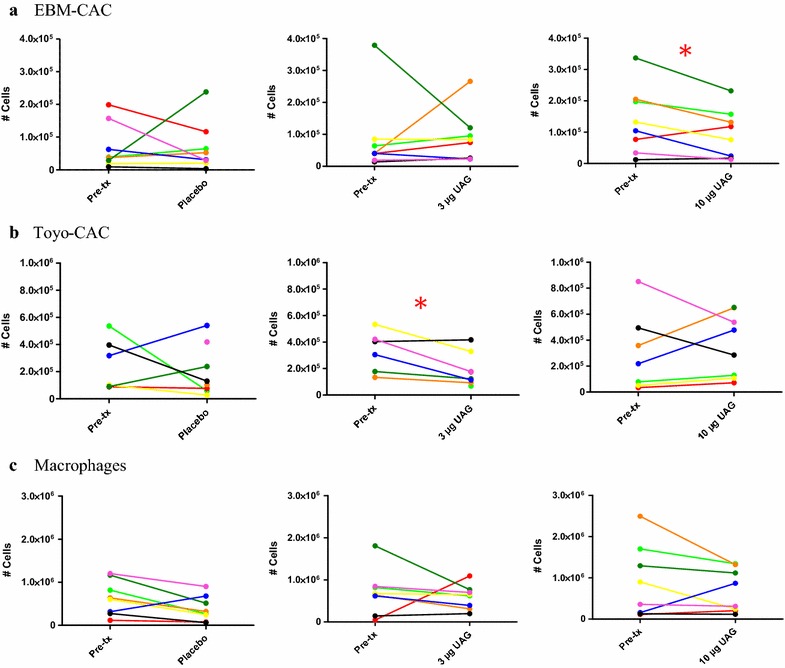
Treatment-related changes in the quantity of obtained EBM-CAC, Toyo-CAC and macrophages. For each patient, the yields of EBM-CAC (**a**), Toyo-CAC (**b**) and macrophages (**c**) derived from PBMC isolated from pre- and post-treatment samples are shown. Each data point represents the average of three individual wells. Quantitative analysis of these data is shown in Table 1

The effect of treatment of patients with 3 or 10 µg UAG/kg/h on CAC and macrophage numbers is shown graphically for each patient in Fig. 4. Table 1 indicates the quantification of these changes, expressed as mean cell count normalized to the mean pre-treatment value. Clearly, the treatment effects were very diverse, and major differences between and among patients were observed. Even the direction of change differed, although for both types of CAC cultures high pre-treatment measurements tended to decrease upon treatment, while low pre-treatment values increased (Fig. 4a, b). Only in two conditions were statistically significant differences observed between pre- and post-treatment samples: treatment with 10 µg/kg/h UAG reduced EBM-CAC to 70% (p = 0.045), while treatment with 3 µg/kg/h UAG caused reduction of Toyo-CAC to 60% (p = 0.038) (Table 1). Enigmatically, however, in both cases treatment with the lower or higher concentration, respectively, did not show a similar trend.

**Table 1:** Treatment-related changes in the yield of CAC and macrophages

Culture	Treatment	Normalized mean cell count post-tx^a^	*p* value^b^
EBM-CAC	Placebo	1.1 ± 1.2	0.219
	3 µg/kg/h	1.0 ± 1.0	0.938
	10 µg/kg/h	0.7 ± 0.6	*0.045*
Toyo-CAC	Placebo	0.8 ± 0.7	0.507
	3 µg/kg/h	0.6 ± 0.4	*0.038*
	10 µg/kg/h	1.2 ± 0.8	0.780
macrophages	Placebo	0.6 ± 0.5	0.053
	3 µg/kg/h	0.9 ± 0.4	0.617
	10 µg/kg/h	1.0 ± 0.8	0.097

For each patient, the baseline values of that specific visit were compared with culture yields after treatment

^a^The mean obtained cell number of pre-tx samples was set at 1; mean post-tx number is expressed relative to this ± SD

^b^Significance was determined by paired t test

For the macrophage cultures, the yield of cells post-treatment tended to decrease in all cases including placebo treatment (Fig. 4c), although single measurements showing increased macrophage yield prevented attainment of statistical significance.

### Assessment of long-term treatment with UAG in mice

**Fig. 5:**
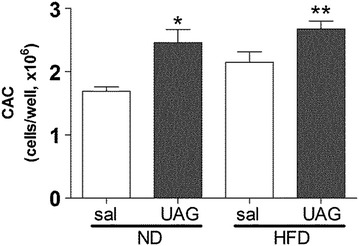
Effect of UAG infusion endothelial progenitor cell recovery from murine bone marrow cell cultures. Mice infused for 4 weeks with UAG fed either a normal or high fat diet during the last 2 weeks of treatment. Bone marrow was then isolated and cultured to generate endothelial progenitor cells (aka. CAC). The data represents the average recovery of cells in three replicate wells of bone marrow cells from three different mice. (*p < 0.05; **p < 0.01 versus ND saline group; ANOVA)

Since short-term treatment of humans with UAG had relatively inconsistent effects, we wanted to determine if longer-term treatment might change this outcome. However, for ethical reasons, long-term treatment of humans is not yet possible. Therefore we used a model of diet-induced obesity in mice. The HFD caused a significant increase in fat mass which was prevented by infusion of UAG, as described in our previous publication [11]. In that study, we also found that UAG infusion prevented the glucose intolerance and insulin resistance that accompanied this increase in fat mass. Testing the effect of 4 weeks of UAG infusion on the differentiation of CAC from bone marrow progenitors showed that infusion of UAG in both ND-fed lean mice as well as mice given a short-term HFD caused a consistent, significant increase in recovery of CAC from bone marrow cultures (Fig. 5).

## Discussion

Previous studies have indicated that T2D is associated with reduced numbers of monocyte-derived CAC [17–19]. Reduced numbers and impaired function of these monocytes in diabetes increased atherosclerosis. Endothelial injury is thought to represent an important step in the initiation and progression of atherosclerotic vascular disease [31]. As mentioned earlier, UAG may have beneficial effects on the vascular remodeling process in T2D patients [20, 21]. Early CAC play a very important role in this process. To further investigate the effect of UAG treatment on CAC, and compare this to macrophage-forming potential, we performed two in vivo studies: a short-term study to assess dose-dependency of UAG effects in T2D patients; and a study to investigate the effects of long-term UAG treatment in mice with decreased insulin sensitivity induced by diet-induced obesity. In humans, we studied the effects of a continuous overnight infusion of UAG or placebo in eight overweight mild type 2 diabetics. We found that short-term infusion of UAG had significant suppressive effects on CAC number at different doses, but this was dependent on differentiation procedure. Our findings are not consistent with those of Togliatto who found that systemic overnight administration of UAG at 3 µg/kg/h (our low dose) prevented diabetes-induced EPC decrease, and increased endothelial progenitor cell mobilization in individuals with T2D [20]. It is currently unknown how UAG interferes with the differentiation of the cells. There is evidence for an Akt-eNOS-MMP9-sKitL pathway that could be involved in release of EPCs into the circulation [20]. The most important explanation for these conflicting results is the heterogeneity in our population. The variation, shown in Fig. 4c, within and among patients is too large to draw firm conclusions; in general, there is no clear effect of the treatment, other than a slight decrease rather than the expected increase of CAC numbers following UAG treatment. An important difference between the studies was that the T2D subjects described in the paper of Togliatto et al. had not been medicated [20]. They were treated with diet alone. In contrast, four of our eight patients were on statins and two used antihypertensive medication. Davignon et al. [32] showed that statins generate potent anti-inflammatory actions and can also improve dysfunctional EPC populations in vitro [33–36]. A potential molecular mechanism of statin action on EPC might involve the PI3 K/Akt pathway [34] and the inhibition of apoptosis [37]. In our population four patients received statin treatment (patient 1, 4, 5 and 8). The statin treatment was put on hold 1 day before testing. With their long half- life, especially of atorvastatin, we can still expect a carry-over effect during the UAG administration in our study. However, a sub-analysis of the statin-using group showed no significant differences between this group and the subjects that did not use statins. This may be caused by the large inter- and intra-individual differences and the small group size. Other studies show that the angiotensin-converting enzyme (ACE) inhibitors such as ramipril [38] and angiotensin II (AT II) inhibitors, like valsartan [39] can increase EPC levels in patients, probably interfering with the CD26/dipeptidyl peptidase IV system. One patient used ramipril, but this treatment was not associated with an increase in the number of circulating CAC. Furthermore, all patients were being treated with metformin, that was stopped 1 day prior to the start of each treatment period. Chronic treatment with metformin in obese patients with T2D did not change ghrelin levels before and after start with metformin [40]. Ida et al., showed in 12 RCTs that there were no significant differences in ghrelin levels [41]. The effect of metformin on UAG is unknown. If there is an effect on UAG, it was similarly in all the patients because metformin was stopped at the same time in all patients. Another explanation for our inconclusive results is the notion that our population with a mean HbA1c of 49 mmol/mol (6.6%) was well regulated. Loomans et al. [30] observed a significant inverse correlation between the number of circulating EPC and HbA1c levels in patients with type 1 diabetes. On average, this population had a 40% reduction in the circulating number of short term cultured EPC, and an HbA1c of more than 8% [30]. These EPC were cultured as described by Murohara [26], described in our study as Toyo-CAC. The question remains whether the number of CAC are affected in our mild diabetics. Loomans et al. obtained similar results in a hyperglycemic mouse model [30]. In this study, it was found that HMG-CoA (3-hydroxy-3-methylglutaryl coenzyme A) reductase inhibitors overcome the effects of hyperglycemia on EPC. Adding this Atorvastatin in vitro to bone marrow differentiation cultures increased the number of EPC and conversely decreased the number of macrophages. Interestingly, this study showed that statin treatment directly increases myeloid EPC generation in vitro from BM precursors derived from mice rendered diabetic by streptozotocin treatment.

In all human samples placebo treatment decreased the yields in macrophage cultures. Maybe this is due to the change of environment. For example the stress of a hospital visit may have caused lower numbers of macrophages. Pre-treatment samples were taken within 1 h after admission and it is known that stress and physical activity increase numbers of circulating leukocytes, including monocytes [27, 42]. Furthermore, the numbers of circulating cells, including monocytes and different populations of EPC, are known to be influenced by circadian variation [43, 44]. Since pre- and post-treatment collection of peripheral blood differ only by 3 h (i.e. 16:00 vs. 13:00 h), it is unlikely that this difference plays a significant role in the observed variability.

An explanation for the high degree of variation observed in the CAC and macrophage cultures can only be speculative at this point. Technical variation may add to the diversity, although we have reason to believe that this contribution is limited. As indicated above, we obtained a very consistent yield of CAC and/or macrophages in a number of pre-treatment samples from individual patients, and these values were acquired in cultures generated over several weeks and at different time points during the study. That leaves a potentially significant contribution of inter- and intra-individual differences induced by inborn as well as environmental conditions as the most likely reason for the observed variability in CAC and macrophage generation.

We have previously reported that a short-term high fat diet causes a doubling in abdominal white fat mass in mice, and significantly impairs insulin sensitivity and glucose tolerance [11]. Using this model we showed that long-term (4 week) UAG infusion at 10 µg/kg/h blocked these effects. Utilizing mice from this study we here also assess the ability of bone marrow progenitors to differentiate into CAC in either vehicle or UAG infused lean or obese mice. Like Togliatto et al. we found that UAG caused an increase in recovery of CAC from differentiated bone marrow cell cultures in obese mice [20]. However, we also find a similar effect in lean mice. In either diet condition, we observed no changes in the generation of macrophages from bone marrow precursors upon treatment with UAG, as reported in our previous study [11]. These data might suggest that longer term treatment in humans may be necessary to observe consistent effects of UAG treatment on CAC, however additional studies are necessary, to confirm this.

## Abbreviations

ACE: angiotensin-converting enzyme
AG: acylated ghrelin
ATII: angiotensinII
BMI: body mass index
CAC: circulating angiogenic cells
EPC: endothelial progenitor cells
GH: growth hormone
GHSR1a: growth hormone secretagogue receptor
GOAT: ghrelin *O*-acyl transferase
HbA1c: glycosylated hemoglobin levels
HFD: high fat diet
HMG-CoA: 3-hydroxy-3-methylglutaryl coenzyme A
ND: normal diet
PBMC: peripheral blood mononuclear cells
PFA: paraformaldehyde
SBM: standard breakfast meal
UAG: unacylated ghrelin
